# The Mediating Role of Self-Compassion in the Relationship Between Perceived Stress and Death Anxiety in Patients with Multiple Sclerosis

**DOI:** 10.3390/healthcare14060743

**Published:** 2026-03-15

**Authors:** Esra Türker, Betül Kılıç, Şeref Demirkaya

**Affiliations:** 1 Department of Nursing, Faculty of Health Sciences, Lokman Hekim University, Ankara 06510, Türkiye; esra.turker@lokmanhekim.edu.tr; 2 Gülhane Vocational School of Health Services, University of Health Sciences, Ankara 06010, Türkiye; 3 Department of Neurology, Gülhane Health Sciences Institute, University of Health Sciences, Ankara 06010, Türkiye; seref.demirkaya@sbu.edu.tr

**Keywords:** multiple sclerosis, self-compassion, perceived stress, death anxiety

## Abstract

Background: Multiple sclerosis (MS) is a long-term and unpredictable condition that can cause considerable psychological distress, including perceived stress and death anxiety. Identifying psychological factors that may mitigate these effects is important for improving the psychosocial well-being of patients with MS. This study aimed to examine the mediating role of self-compassion in the relationship between perceived stress and death anxiety in patients with MS. Methods: This cross-sectional, descriptive, and correlational study included 169 Turkish patients diagnosed with MS between October 2024 and April 2025. A regression-based mediation analysis using the Hayes PROCESS macro with bootstrapping was conducted to assess the mediating role of self-compassion. Results: Death anxiety scores were positively but weakly correlated with perceived stress scores (r = 0.172, *p* = 0.026). Perceived stress scores were strongly and negatively correlated with self-compassion scores (r = −0.704, *p* < 0.001), whereas self-compassion scores showed a weak-to-moderate negative correlation with death anxiety scores (r = −0.287, *p* < 0.01). In the mediation model, perceived stress significantly predicted self-compassion (B = −0.087, *p* < 0.001), and self-compassion significantly predicted death anxiety (B = −1.758, *p* < 0.001). The direct effect of perceived stress on death anxiety was not statistically significant (B = −0.058; *p* = 0.344), whereas the indirect effect was significant (B = 0.153; 95% CI [0.079, 0.232]). The total effect was also significant (B = 0.095; *p* = 0.036). Conclusions: The findings indicate that self-compassion mediates the relationship between perceived stress and death anxiety in patients with MS. Higher levels of self-compassion were associated with lower levels of perceived stress and death anxiety, suggesting that self-compassion may function as an important psychological resource in coping with disease-related stress and death-related concerns. From a clinical and nursing perspective, integrating strategies that support self-compassion within holistic care may contribute to improving the psychosocial well-being of patients with MS.

## 1. Introduction

Multiple sclerosis (MS) is an autoimmune, demyelinating, and neurodegenerative disease of the central nervous system and is the leading cause of non-traumatic neurological disability in young adults [1]. The disease causes symptoms of varying severity depending on which areas of the brain or spinal cord are affected and has multidimensional effects on physical, cognitive, and psychosocial functioning [2]. In addition to physical disability, many psychosocial symptoms, such as stress, fatigue, cognitive difficulties, sleep disorders, and a decline in quality of life, are also seen in patients with MS [3]. Although psychosocial symptoms significantly impact patients’ daily lives, they are often overlooked in routine patient follow-ups, where physical disabilities are prioritized [4]. However, it is vitally important to monitor mental health as seriously as physical symptoms and to provide comprehensive support. 

Despite the development of numerous treatment options to alter the course of MS, a cure that completely eliminates the disease has not yet been found [5]. The unpredictability of disease progression and the areas it may affect, along with the uncertainty of recovery after diagnosis, continue to place a significant burden on patients with MS [4]. Factors such as the uncertainty caused by the disease, loss of independence, and decreased social functioning have been shown to increase anxiety and stress levels in conjunction with physical disability [3,6].

Death anxiety is a psychological concept encompassing emotional responses, such as worry, fear, and discomfort, arising from contemplation of one’s own or a loved one’s mortality [6].

The extant literature indicates that death anxiety exerts a deleterious effect on an individual’s physical and mental well-being, potentially giving rise to conditions such as depression and anxiety [3,6]. Consequently, given the absence of a definitive treatment for MS and the unpredictable nature of its clinical course, it is anticipated that death anxiety levels will be elevated in patients with MS compared to those in the general population [5]. Therefore, it is crucial to investigate the effects of perceived stress and death anxiety on psychological well-being in patients with MS [7]. In this context, the notion of self-compassion can serve a protective function, aiding individuals in their ability to cope with adversity [8]. Self-compassion is defined as the ability to endure painful and distressing emotions with equanimity, approach oneself with benevolence and understanding, accept oneself with compassion rather than condemn oneself for perceived failures, and perceive negative experiences as inherent aspects of the human condition [9]. The cultivation of self-compassion has been demonstrated to be advantageous in circumstances involving challenging life events, such as chronic illness, or when feelings of inadequacy emerge due to physical activity limitations resulting from a disability [8]. Research has shown that self-compassion is positively correlated with positive variables such as self-acceptance, life satisfaction, social interest, awareness, autonomy, personal development, happiness, and optimism, and negatively correlated with emotions such as anxiety and fear of death [10,11]. Therefore, it is important to assess death anxiety when assessing self-compassion. Defining and understanding death anxiety in patients with MS is not merely a matter of measuring a psychological variable; it is a clinically significant area of assessment that contributes to a more comprehensive explanation of depression, perceived stress, and emotional adjustment processes. Determining patients’ levels of self-compassion and developing individualized care and treatment plans accordingly is particularly important for strengthening the holistic approach to MS care. This can support patients in adopting a more accepting attitude toward their experiences while participating in daily life activities, and it strengthens their psychological adjustment [12].

Research on self-compassion in patients with MS is limited. In addition, levels of self-compassion may be influenced by the sociocultural context in which individuals live [11]. Therefore, determining the relationships between perceived stress, death anxiety, and self-compassion in patients with MS is important for timely intervention in disease management and the development of support programs [7,10]. Accordingly, this study aimed to examine the mediating role of self-compassion in the relationship between perceived stress and death anxiety in Turkish patients with MS. The following four hypotheses were developed and tested:

(1) Perceived stress is positively associated with death anxiety in patients with MS.
(2) Self-compassion is negatively associated with perceived stress in patients with MS.
(3) Self-compassion is negatively associated with death anxiety in patients with MS.
(4) Self-compassion mediates the relationship between perceived stress and death anxiety in patients with MS.

## 2. Materials and Methods

### 2.1. Study Design

This study was carried out as a cross-sectional, descriptive, and correlational study between October 2024 and April 2025.

### 2.2. Setting and Sample

The study was conducted with MS patients visiting the neurology outpatient clinic and under medical supervision. To determine the sample size, a priori power analysis was performed using the WebPower R package [13], based on the medium effect size values defined by Fritz and MacKinnon (2007) (α = β = 0.39) [14]. Under the conditions of α = 0.05 and 1 − β = 0.90, the minimum sample size was calculated as n = 140. Non-probability convenience sampling was used to select participants. The study was completed with n = 169 participants. The inclusion criteria for the study were as follows: participants were volunteers who could read and write Turkish, were 18 years of age or older, had been diagnosed with MS, belonged to the relapsing–remitting MS or progressive MS (primary/secondary) group, and did not have a diagnosed mental illness that impaired communication. Exclusion criteria were severe vision and hearing problems and being in the active phase of the disease.

### 2.3. Data Collection Tools

#### 2.3.1. Patient Form

This form consists of 11 questions developed by researchers using the relevant literature [6,8,10,11] and provides information about the patient’s age, sex, marital status, education level, employment status, duration of illness, presence of MS in the family, type of MS, Expanded Disability Status Scale (EDSS) score, and duration of physical symptoms.

#### 2.3.2. Perceived Stress Scale

The scale is a tool used to assess the subjective experience of stress in individuals. The scale in question was developed by Cohen et al. and subsequently adapted into Turkish by Bilge et al. [15,16]. The scale is a 5-point Likert scale with two subscales: perceived stress and perceived coping. The total score ranges from 0 to 32. A high total score indicates a high level of perceived stress. The Cronbach’s alpha value of the scale is 0.81 [16]. The Cronbach’s alpha value in this study was 0.84.

#### 2.3.3. Self-Compassion Scale

Neff’s scale has been adapted into Turkish by Akın et al. [17,18]. The scale is a measurement tool that evaluates characteristics related to the subdimensions of self-compassion. It is based on the individual providing information about themselves. The Self-Compassion Scale is a 26-item instrument with a 5-point Likert-type rating scale. As the total score on the scale increases, self-compassion increases. The Cronbach’s alpha value of the scale was determined to be 0.94 [18]. The Cronbach’s alpha value in this study was 0.95.

#### 2.3.4. Death Anxiety Scale (Das)

Developed by Templer and Ruff and adapted into Turkish by Akça and Köse, the scale consists of 15 items and is organized as a binary Likert scale with true/false responses [19,20]. Correct responses are given 1 point, while incorrect responses are not scored. As the score increases, death anxiety increases. Individuals with an average score of 7 or above on the scale are considered to have high levels of death anxiety. The Cronbach’s alpha value of the scale was found to be 0.75 [20]. In this study, the Cronbach’s alpha value was found to be 0.81.

### 2.4. Data Collection

Informed consent was obtained from participants before administering the data collection form. Participants who signed the consent form were given data collection forms to fill out themselves. Participants completed the forms in a quiet room. The average time spent completing each data collection form was 15–20 min. The study was reported according to the Strengthening the Reporting of Observational Studies in Epidemiology (STROBE) guidelines [21].

### 2.5. Ethical Approval

Prior to this study, the researcher obtained written permission from the Research Ethics Committee of the University (Decision no: 2024/214; Code no: 2024203). In addition, the researchers informed the participants verbally and in writing about the nature and purpose of the study and obtained their consent before starting the study. The Helsinki Declaration was adhered to during the research process.

### 2.6. Data Analysis

Statistical analyses were performed using IBM SPSS Statistics 26.0 (SPSS Inc., Chicago, IL, USA). The normality of numerical variables was examined using visual (histograms and probability plots) and analytical methods (Shapiro–Wilk test, skewness and kurtosis values, and coefficient of variation). Descriptive statistics are presented as frequency and percentage for nominal and ordinal variables and as median and 25th–75th percentiles or mean and standard deviation for numerical variables. The relationship between variables was examined using the Pearson correlation test [22,23]. A mediation model (Model 4) was created using the Hayes PROCESS macro to investigate the complex relationships between perceived stress, self-compassion, and death anxiety [24]. In the mediation model, “death anxiety” was the dependent variable, “perceived stress” was the independent variable, and “self-compassion” was the mediator variable. Age, gender, and disease duration were included as control variables in the models. Cases where the Type 1 error rate was below 5% were considered statistically significant.

## 3. Results

Among the 169 MS patients who participated in our study, 86.98% were female, with an average age of 38.05 ± 10.04 (mean ± SD). A subsequent analysis of the disease phenotype revealed that 34.91% of patients were diagnosed with relapsing–remitting MS (RRMS), while no information was provided about the disease phenotype in the remaining patients. However, a subsequent analysis of the medications used by these patients indicated that most of them also had RRMS. The mean disease duration of the patients participating in our study was 7.32 years, with a standard deviation of ±6.01 years. According to the EDSS scores based on the participants’ self-reports, 93% of the patients had a score between 0 and 4, 7.1% had a score between 4 and 6, and there were no patients with an EDSS score of 6 or above. Among the patients diagnosed with MS who were included in the study, 58.58% were married, and 78.7% had received a university education. Furthermore, the vast majority of the participants resided in urban areas. Most of the participants (79.88%) did not report a family history of MS (Table 1).

The mean total scores on the Perceived Stress Scale for the MS patients participating in the study were 13.66 ± 6.15 (1–27). The total score they received on the Death Anxiety Scale was 6.96 ± 3.71 (0–14), and the total score they received on the Self-Compassion Scale was 3.32 ± 0.79 (1.46–4.85). The findings suggest that individuals with MS exhibit moderate levels of perceived stress and death anxiety, while their self-compassion levels are relatively higher within the scale range.

Correlation analysis conducted to examine the relationships among the study variables revealed a weak positive association between total death anxiety and perceived stress scores (r = 0.172; *p* = 0.026). A strong negative correlation was observed between total self-compassion and perceived stress scores (r = −0.704; *p* < 0.001). In addition, a weak negative correlation was identified between total self-compassion and death anxiety scores (r = −0.287; *p* < 0.001) (Table 2).

**Figure 1:**
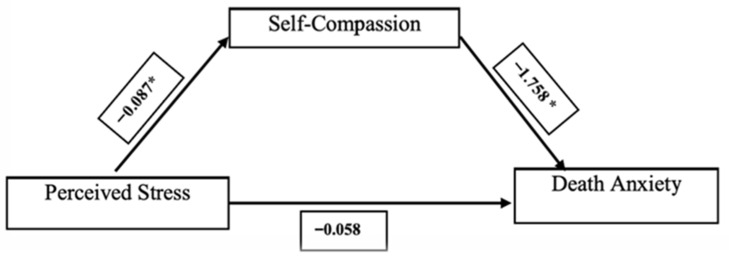
The mediation model showed the mediating role of self-compassion in the relationship between stress and death anxiety. Non-standardized regression coefficients are shown for the relationships between the variables. * *p* < 0.001.

In the mediation model, perceived stress significantly predicted self-compassion (B = −0.087; *p* < 0.001), and self-compassion significantly predicted death anxiety (B = −1.758; *p* < 0.001). The direct effect of perceived stress on death anxiety was not statistically significant (B = −0.058; *p* = 0.344), whereas the indirect effect was significant (B = 0.153; 95% CI [0.079, 0.232]). The total effect was also significant (B = 0.095; *p* = 0.036). These findings indicate that self-compassion mediates the relationship between perceived stress and death anxiety. Because the direct and indirect effects were in opposite directions, this pattern is consistent with an inconsistent (competitive) mediation model, suggesting a suppression-type effect (Table 3; Figure 1).

## 4. Discussion

To our knowledge, this study is the first to analyze whether self-compassion plays a mediating role in the relationship between perceived stress and death anxiety among people with MS in Türkiye. The mean total scores on the perceived stress scale for participants with MS were found to be moderate. In a similar study, more than half of the participants also reported moderate levels of perceived stress [25]. Psychological stress has been demonstrated to exacerbate MS symptoms by exerting deleterious effects on the immune system. Psychological stress has been identified as a significant contributor to the exacerbation of fatigue, a prevalent symptom that substantially impacts the quality of life of individuals diagnosed with MS. As indicated in a study by Novak et al., patients suffering from MS who exhibited elevated stress levels reported experiencing significantly higher levels of fatigue in comparison to those demonstrating low stress levels [3]. It is imperative for individuals diagnosed with MS to develop effective coping mechanisms to manage the stress associated with their illness. This stress can hinder their ability to perform daily activities and potentially accelerate the progression of the disease. Given the limited efficacy of treatment options available for managing MS, effective nursing care entails a collaborative approach with the patient, a disease-specific care plan, and a comprehensive assessment of the patient’s stress levels [26].

Psychosocial problems in patients, a lack of social support, and feelings of loneliness in their social environment can make thoughts of death more threatening [27]. The present study also examined death anxiety in individuals with MS and found it to be moderate. In another study, it was found that the 56 patients participating in the study had a moderate level of fear of death and that MS patients with high EDSS scores had greater anxiety about death [7]. The average death anxiety scores of MS patients included in this study can be attributed to three factors. First, the patients exhibited low disability scores, indicating a lack of dependence on assistive devices such as crutches or canes for mobility. Second, the absence of patients who were wheelchair-bound or bedridden contributed to the findings. Furthermore, the MS patient association in our region conducts regular patient–physician meetings to discuss the characteristics of the disease and the problems faced by patients. One of the issues raised at these meetings is death related to the disease, which may have raised awareness in this area among our study group. It is imperative to acknowledge the prevalence of death anxiety, a significant psychological condition, among individuals diagnosed with MS. This condition is frequently disregarded in clinical settings, underscoring the need for enhanced awareness and management strategies to address its impact on patient well-being. It is the position of the present authors that the assessment and management of death anxiety should be considered an integral component of MS treatment. In this regard, it is recommended that physicians treating patients, nurses providing care, and patient associations provide information and support to patients.

MS is a chronic condition that places significant demands on patients’ physical and mental well-being. During this process, the cultivation of self-compassion has been shown to facilitate more robust psychological adjustment by enabling individuals to develop a more supportive, compassionate, and understanding attitude toward themselves [10]. In the present study, the self-compassion status of patients was examined, and it was found that their self-compassion scores were high. A study of the literature revealed that individuals diagnosed with MS exhibited self-compassion scores that surpassed the mean average, a finding that aligns with the results of our study [28]. The present study hypothesizes that structured psychological interventions designed to cultivate self-compassion may have a complementary role in the treatment of MS. Such interventions have the potential to facilitate a shift in patients’ perspectives towards their illness, characterized by increased acceptance and compassion. Furthermore, these interventions may enhance patients’ ability to effectively cope with negative thoughts.

The initial hypothesis of this study posited that perceived stress would be positively associated with death anxiety in patients with multiple sclerosis (MS). The findings supported this hypothesis, revealing a weak positive correlation between perceived stress and death anxiety. These results are consistent with those of a previous study that reported an association between perceived stress and death anxiety in individuals with MS [29]. The fact that MS, a chronic disease, typically manifests initially in young adulthood, exhibits unpredictable attack processes, and may cause physical symptoms leading to disability contributes to an increase in patients’ perceived stress levels and death anxiety [30]. D’Anca et al.’s study indicates that women exhibit higher levels of perceived stress and greater death anxiety than men in the context of chronic diseases [31]. Given the higher prevalence of MS in the female population, the findings of this study carry considerable significance. In the present study, the fact that women constituted the majority of the sample was consistent with the extant literature, which reported moderate levels of perceived stress [31].

The second hypothesis proposed that self-compassion would be negatively associated with perceived stress in patients with MS. The findings supported this hypothesis, revealing a strong negative correlation between self-compassion and perceived stress levels. This result is consistent with previous research indicating that individuals with higher self-compassion tend to experience lower levels of perceived stress [32]. In chronic conditions such as MS, self-compassion is considered an important psychological resource that may facilitate adaptive coping with disease-related physical symptoms and promote active engagement in treatment and care processes. Additionally, these individuals are less likely to blame themselves for the physical challenges they face and may utilize social support mechanisms more effectively, which may be associated with improved quality of life [32]. Previous studies have suggested that self-compassion increases psychological flexibility, reduces stress levels, and supports adaptation to chronic illness [11,33]. In this context, interventions aimed at strengthening self-compassion may contribute to psychological well-being, disease management, and social integration.

Another hypothesis of this study was that self-compassion would be negatively correlated with death anxiety in patients with MS. The findings supported this hypothesis, indicating an inverse relationship between self-compassion and anxiety about death. This result is consistent with the findings of previous studies. Individuals with chronic illnesses reportedly experience higher levels of death anxiety than healthy individuals [29]. Furthermore, prior studies have indicated that patients with MS without clinically significant anxiety symptoms may still report elevated death anxiety [7]. Additionally, Abdollahi et al. found that MS patients with more positive self-perceptions experienced lower levels of death anxiety [29]. Taken together, these findings suggest that self-compassion may function as a protective psychological resource that facilitates coping with illness-related existential distress.

In the structural equation model testing the final hypothesis, self-compassion significantly mediated the relationship between perceived stress and death anxiety in patients with MS. The pattern of coefficients was consistent with an inconsistent mediation (suppression) model, in which the indirect and direct effects operated in contrast to each other. Although disease-modifying treatments are available, MS remains an incurable condition, and the cumulative physical and psychological burden of the disease may increase perceived stress and death-related concerns [29]. In this context, higher levels of self-compassion appear to function as a psychological buffer, being associated with lower perceived stress and reduced death anxiety. These findings suggest that self-compassion may be a protective psychological resource that enhances patients’ capacity to cope with illness-related existential distress.

## 5. Conclusions

This study examined the mediating role of self-compassion in the relationship between perceived stress and death anxiety in patients with multiple sclerosis, using a regression-based mediation analysis implemented through the Hayes PROCESS macro with bootstrapping. These findings indicate that self-compassion is an important psychological factor associated with perceived stress and death anxiety in patients with MS. These results suggest that higher levels of self-compassion may support patients’ ability to cope with stress and death-related concerns during the disease process. From a clinical and nursing perspective, adopting a holistic approach that considers psychological resources, such as self-compassion, may complement pharmacological treatment and contribute to supportive care for patients with MS. In this context, nurses, who are closely involved in the long-term care and follow-up of patients with chronic diseases such as MS, are in a key position to assess patients’ psychological needs and incorporate supportive strategies that foster self-compassion within holistic nursing care. The mediation model in this study was estimated using observed composite total scores rather than latent subscale indicators; future studies should test latent variable models incorporating subscale indicators to further explore the structural relationships among these constructs.

Additionally, future research should examine how self-compassion influences the relationship between perceived stress, death anxiety, and other outcome variables in these patients and whether these relationships vary across different populations and cultural contexts. Such studies may provide further insights into developing interventions to improve the quality of care for patients with MS.

## 6. Limitations

This study had several limitations that should be considered when interpreting the findings. First, the cross-sectional design precludes causal inferences regarding the relationships between perceived stress, self-compassion, and death anxiety. Second, the use of self-report measures may have introduced response bias and shared method variance. Third, the mediation model was estimated using observed composite total scores rather than latent variables, which may limit the ability to account for measurement errors. In addition, the sample was recruited from a single clinical setting, which may restrict the generalizability of the results to the broader MS population. Finally, although relevant covariates were controlled for, other unmeasured psychological or clinical factors may have influenced these relationships. Future longitudinal and multicenter studies using latent variable modeling approaches are recommended to confirm and extend these findings.

## Figures and Tables

**Table 1:** Characteristics of participants.

Characteristics of Participants		
Age: 38.05 ± 10.04		
Diagnosis time: 7.32 ± 6.01		
	n	%
Sex		
Female	147	86.98
Male	22	13.02
Education Status		
High school	36	21.30
University and above	133	78.70
Marital Status		
Single	70	41.42
Married	99	58.58
Place of Residence		
Province	156	92.31
County	13	7.69
Family history of MS		
Yes	34	20.12
No	135	79.88
Information About MS Disease		
Yes	76	44.97
No	93	55.03
MS type		
Undetermined	65	38.46
Relapsing–remitting type	59	34.91
Primary progressive	28	16.57
Secondary progressive	17	10.06
EDSS		
<4	118	69.82
4–6	39	23.08
6≥	12	7.1
Duration of Physical Difficulty		
0–1 year	46	27.22
2–4 years	65	38.46
More than 5 years	58	34.32

n: frequency, %: percent.

**Table 2:** The relationship between death anxiety, self-compassion, and perceived stress.

		Perceived Stress	Death Anxiety
Death anxiety	r	0.172	-
	*p*	0.026	-
Self-compassion	r	−0.704	−0.287
	*p*	<0.001	<0.001

Pearson correlation analysis was applied.

**Table 3:** The mediating effect of self-compassion in the relationship between perceived stress and death anxiety.

Model Pathways		B	95% CI	t	*p*
Perceived Stress → Self-Compassion		−0.087	−0.100/−0.073	−12.595	<0.001
Self-Compassion → Death Anxiety		−1.758	−2.727/−0.788	−3.579	<0.001
Perceived Stress → Self-Compassion → Death Anxiety					
	Direct effect	−0.058	−0.178/0.062	−0.950	0.344
	Indirect effect	0.153	0.079/0.232	-	-
	Total effects	0.095	0.006/0.184	2.114	0.036

The model is adjusted for sex, age, and disease duration. Bootstrap N = 5000. B: unstandardized coefficients, CI: confidence interval.

## Data Availability

The data supporting the findings of this study are available upon request from the corresponding author. The data are not publicly available because of privacy or ethical restrictions.
